# Critical reflections of postgraduate researchers on a collaborative interdisciplinary research project

**DOI:** 10.1057/s41599-022-01494-w

**Published:** 2023-01-06

**Authors:** Ben Purvis, Hannah Keding, Ashley Lewis, Phil Northall

**Affiliations:** 1 Sheffield University Management School, University of Sheffield, Sheffield, UK; 2 Fraunhofer-Informationszentrum Raum und Bau, Stuttgart, Germany; 3 Independent Scholar, Edinburgh, UK; 4 Centre for Regional Economic & Social Research, Sheffield Hallam University, Sheffield, UK

**Keywords:** Education, Science, technology and society

## Abstract

By employing a retrospective collaborative autoethnographic approach, this work aims to better understand how an interdisciplinary context shaped the authors’ experiences of British academia during their Ph.D research. The authors bring together their individual observations and experiences to collectively interrogate and critically reflect on their position as postgraduate researchers (PGRs) on a collaborative interdisciplinary research project. These reflections are taken as a lens through which to interrogate the contemporary British university. Pre-existing tensions within the academy are characterised as ‘asymmetries’ along dimensions of risk, disciplinary hierarchy, and knowledge. It is argued that the authors’ experience of uncertainty and precarity as junior academics stems principally from pre-existing structures within British academia, rather than the interdisciplinary environment in which they were immersed. By emphasising the role of the successfully trained doctoral candidate as an outcome itself, it is argued that indicators of success can be reframed, shifting the power asymmetry to place greater value on PGRs within the neoliberal academy. Highlighting the ambiguity of their convergent and divergent personal experiences, the authors suggest there is a need for a greater focus on the contested role of the PGR within the contemporary university system.

## Introduction

The contemporary British university system represents a landscape of asymmetries. From the disparity in pay and security between senior managers and hourly paid staff (Rings, 2021), the system reproduces existing inequalities and the politics of exclusion along dimensions of gender, race, and class (Brooks et al., 2020; Doharty et al., 2021; Wilson et al., 2021). Further asymmetries are seen within a funding landscape that favours large grants over small, and the established academic over the early career academic (ECA) (Aagaard et al., 2020; Bol et al., 2018), as well as the systematic undermining of departments, disciplines, and programmes deemed not sufficiently profitable or failing to generate ‘impact’ (Chubb and Reed, 2018; University and College Union, 2012).

As postgraduate researchers (PGRs), we pursued our Ph.Ds as members of a large interdisciplinary collaborative research project composed of scholars from physical, computational, economic, and social sciences (2015–2020). This experience represented our induction into the academic system and offered unique perspectives across and within disciplinary traditions. Within this work our central research question seeks to better understand how and to what extent the interdisciplinary nature of this environment shaped us as researchers in our formative years. In exploring these questions we offer a lens from below, linking our individual and collective experiences as PGRs on a collaborative interdisciplinary research project to wider tensions within British academia. As well as exploring this journey, our secondary aim is to reframe ‘success’ in such projects around the narratives of the researchers situated within them. Our work primarily contributes to the literature on interdisciplinarity, with secondary relevance to the fields of critical university studies and science & technology studies. The novelty of this contribution lies within its retrospective collective autoethnographic approach, drawing directly on the lived experience of researchers conducting their Ph.Ds within an interdisciplinary group environment.

We begin in section ‘The place of the interdisciplinary PGR within the British University’ by surveying the landscape of the contemporary British university system in which we were situated, reviewing the literature on the neoliberal academy, the rise of interdisciplinary research, and the role of the PGR. In doing so, we set the scene for the landscape in which we pursued our Ph.Ds, as well as grounding our later observations within the literature. section ‘Methodology & context’ illustrates our methodological foundations, outlining how we draw on retrospective collective autoethnography and critical reflection to collate and analyse our data in relation to our research questions. We provide detail of the contextual specificity of ‘The Project’ and discuss limitations of such an approach. section ‘Critical reflections of our contextual experience’ presents our critical reflections in the form of three asymmetries relating to risk, disciplinary hierarchy, and knowledge. Through these we explore how the specificities of our interdisciplinary environment shaped our experiences as PGRs. Section ‘Discussion’ draws together these three themes in light of the afore-analysed literature, addressing our research questions by considering our experiences in relation to the wider tensions within British academia. Within this discussion, we go on to consider how the PGR experience might be rewritten and reframed to centre on care for the individual and celebrate personal success. We conclude in section ‘Outlook’ with some final considerations of our findings and how future research agendas might build upon them.

## The place of the interdisciplinary PGR within the British University

To understand how the interdisciplinary environment we were situated in as PGRs shaped our experiences of the academic landscape, it is first necessary to set the scene with reference to the relevant literature. This is done so across three relevant dimensions. We first contextualise the British University in terms of the ‘neoliberal academy’ as well as outlining the position of PGRs within this context. Second, we outline the literature on interdisciplinarity and associated research practices, including how ‘success’ has been conceptualised within this regime. Finally, section ‘Navigating this space as a PGR’ assesses the literature on the PGR experience, particularly within interdisciplinary environments.

### Precarity and risk in the neoliberal academy

The diffusion of a neoliberal logic into the university sector, characterised by Harvey (2005) through its emphasis on entrepreneurial individualism and subservience to market forces, has received significant attention in the anglophonic research landscape, particularly under the regime of austerity imposed post-2008 (Ivancheva et al., 2019; Ross et al., 2020; Vernon, 2018). Often enabled through the ‘trojan horse’ of real-estate management (Shen, 2020), this approach results in “changes in organisational culture and a power shift from teaching and research professionals to accountants, real-estate developers, financiers and their ilk” (Engelen et al., 2014: p. 1072). Within the UK context this has manifested in the form of rising tuition fees, outsourced services and staff, rent extraction, the proliferation of metrics such as the Research Excellence Framework (REF), and league tables emphasising ‘student experience’ and ‘employability’ (Christie, 2017; Holbrook, 2017; McCann et al., 2020). Necessarily, this has led to a growing body of critical literature on the consequences of this neoliberal turn, and its effects on the various actors within the academic machine, from feminist, queer, anti-racist, and decolonial perspectives (see e.g., Bartram, 2020; Bhambra et al., 2018; Brooks et al., 2020, Doharty et al., 2021), as well as intersections with the widespread precarious employment within the sector (Kezar et al., 2019, Rings, 2021).

The instability of this regime is, at the time of writing, being uncovered in a new light through the ongoing impacts of the COVID-19 pandemic (Urban ECA Collective et al., 2022). Studies reporting high levels of anxiety, stress, and depression among undergraduate students, PGRs, and university staff (Marelli et al., 2021; Wang and Zhao, 2020; Wray and Kinman, 2021) add to an already burgeoning literature surrounding a mental health crisis within academia, with existing studies suggesting that between a third to a half of graduate researchers experience mental health issues (Evans et al., 2018; Levecque et al., 2017). Within the UK, the panic that ensued in response to a forecast drop in student numbers, realised through hiring freezes, redundancies, and institutional austerity across the sector emphasised an increasingly parlous funding model, heavily reliant on student fees especially from international markets (Ahlburg, 2020; Findlay et al., 2017). Further, the impact on additional revenue streams founded on rent extraction, such as university owned accommodation, conference venues, and catering facilities demonstrate the extent to which the erosion of public funding, has left a sector unable to cope with market shocks (Burki, 2020; Cundy, 2020; Kınıkoğlu and Can, 2020). In the absence of government bailouts, university management has had to negotiate the trade-off between the health and safety of their staff and students with financial bottom lines, invariably prioritising the latter. The dominant media narratives surrounding these ongoing crises have focused on the student as a consumer, and higher education as a commodity, with universities failing to provide ‘value for money’ through the shift to online teaching (Adeluwoye, 2020; BBC, 2020). Less media attention has been given to the treatment of postgraduate researchers, many of whom have lost income through cuts to graduate teaching positions (Corona Contract, 2020), and have been refused funded extensions to circumvent the significant delays and impacts that have affected many projects (Dickinson, 2020; Munro and Heath, 2021; Quinn, 2020).

Within the context of the British neoliberal university, widespread precarity is seen in the form of increasingly outsourced cleaning, maintenance, and security staff, as well as hourly paid lecturers, and staff on fixed-term contracts (Rings, 2021). Here, the PGR occupies an uncertain place, while many may be employed on various ‘graduate teaching assistant’ contracts, supporting teaching work alongside their Ph.D studies, the Ph.D itself is not classed as employment. Tuition fees are often covered by external funders, including research councils and charities, who may also provide a stipend, i.e., a grant for living costs, which is paid to the researcher monthly, though many PGRs are self-funded. The role of the PGR in the UK thus typically affords none of the associated rights, which come with employment status such as minimum wage and guaranteed parental leave, while limiting access to financial products such as mortgages (Brown, 2004). This status lies in contrast to many other countries in Western Europe, including Sweden, Norway, and Denmark where doctoral PGRs are contracted employees, and Switzerland, the Netherlands, and Germany where a large number hold employment status (Cornell, 2020).

Alongside the neoliberal turn within the academy, the last decades have witnessed a transformation of knowledge production, from disciplinary-oriented research to applied problem-oriented cross-disciplinary research, referred to by Gibbons (1994) as Mode-1 and Mode-2, respectively. This shift has been accompanied by the ‘impact agenda’, which has created a competitive regulatory landscape for research funds through policy makers’ desire to impose accountability in the allocation of public funds (Holbrook, 2017). While this has led to an increase in cross-disciplinary research to address ‘wicked problems’ (Gibson et al., 2019), it has also been linked to the emergence of ‘Mode-2 universities’ whose missions place “emphasis on research and the overvaluation of publications” (Sousa, 2011: p. 63), adopting the logic of the ‘publish or perish’ model of academic career progression (van Dalen and Henkens, 2012). Across the UK context an entrenched hierarchy exists, strongly linking a notion of prestige to how long ago a university was granted its charter; this asymmetry reproduces existing societal inequalities and is reinforced by the disproportionate allocation of research funds to these ‘elite’ institutions (Boliver, 2015; Croxford and Raffe, 2015).

### The rise of interdisciplinary research

A natural progression of Mode-2 problem-oriented research, is a rise in popularity and notoriety of ‘interdisciplinary’ research. Funding bodies reward and fund projects that bring multiple disciplines together under one project (Rylance, 2015; Science Europe, 2012). The cited motivation for increasing disciplinary collaboration is that multiple perspectives can lead to more ‘holistic’ research and solutions, which are particularly necessary for ‘complex’ problems such as sustainability (Frank, 2017; Lam et al., 2014). Such research is therefore associated with having higher impact (Davé et al., 2016; Nurse, 2015). As demonstrating research impact has become a key funding requirement for many funders (Kidd et al., 2021; Science Europe, 2017; UKRI, 2021) interdisciplinarity offers a pathway to cement this relevance.

Within this context, authors working on interdisciplinary projects have documented various challenges and best practices of collaboration (Pischke et al., 2017; Trussell et al., 2017). The most common difficulties articulated include communication and the challenge to be understood (Albert et al., 2009; Darbellay, 2015), the opaque definition(s) of interdisciplinarity (Cooper, 2013), compounded time constraints (Datta, 2018), power asymmetries (MacMynowski, 2007), and structural barriers preventing cohesive knowledge ‘integration’ (Lyall, 2019). Another core challenge relates to assessing the impact of the ‘interdisciplinary’ efforts on the quality of research, and is exacerbated by the lack of indicators to gauge such efforts as ‘successful’ (Lewis, 2021). The myriad of definitions and a lack of uniform usage to describe interdisciplinarity contributes to making what is judged ‘successful’ hard to pinpoint (Cooper, 2013). Lam et al. (2014) discuss how the term interdisciplinary can broadly encompass multi-, inter- and transdisciplinarity, while Klein (2010) categorises multi-, inter-, and transdisciplinarity, respectively, on a sliding scale according to their increasing level of disciplinary integration.

What could be seen as a ‘successful’ multidisciplinary project might not qualify as the more desirable ‘interdisciplinary’ project in the eyes of some researchers (Aboelela et al., 2007). In addition, what could be considered a ‘successful’ outcome for one researcher might not translate to an equal success for another researcher from a different discipline. For example, being cited on a multi-authored paper contributes more to a natural scientist’s career than a social scientist, as social scientists are judged more heavily by single authored publications (Helgesson and Eriksson, 2019). As it can take longer for interdisciplinary projects to take off, they can often be viewed as ‘unproductive’ (Goulden et al., 2017), because so much lead time is required for disciplinary experts to ‘get to grips’ with the project. This creates a paradox as more time is needed for interdisciplinary collaborations leading to potentially fewer ‘outputs’, yet interdisciplinarity is expected to be more impactful due to its ‘problem-solving’ approach (Goyette, 2016).

To confront some of these challenges it is helpful to think about how we define and understand the ‘discipline’. As the term itself suggests, often authors consider interdisciplinarity to be the borders in-between, or the overlapping space of two or more disciplines (Callard and Fitzgerald, 2015; Castán Broto et al., 2009). This narrative uses the discipline as a reference point for identifying interdisciplinarity, assuming it to be a pre-existing or static notion. An alternative narrative places interdisciplinarity as the countermovement against the historical process of disciplines becoming more specialised and posits that it is not a new or singular phenomenon (Castán Broto et al., 2009). Papers that track and study the history of disciplines point out that some subjects, which are now considered separate originated from a singular mode of study (Weingart, 2010). Barry et al. (2008) argue that interdisciplinarity is not recent, and that rarely have research studies taken place in a singular space or discipline.

As more disciplines continue to emerge through differentiation (Stichweh, 2003) and specialisation (Casadevall and Fang, 2014) of knowledge, interdisciplinarity becomes a constantly moving target and is impossible to describe and study as a single state over time (Wagner et al., 2011). Such narratives recognise the diversity within disciplines as well as the dynamic changing nature of disciplines. This dynamic nature makes it difficult to demarcate the boundaries between a disciplinary study and an interdisciplinary study. Gieryn (1983) recognises this paradox of defining boundaries between disciplines and identifies the tacit processes, negotiations and language used by scientists to demarcate their field of expertise or knowledge from other forms of knowledge production. The disciplinary boundaries created by research practitioners extend beyond epistemological and ontological understanding, to the cultural and performative practices of a discipline enforced by disciplinary core groups (Becher, 1989). For example, disciplines adopt certain publishing practices, outline acceptable language and vocabulary, and establish rites of passage for trainees in their discipline.

### Navigating this space as a PGR

The above context sets the stage onto which the PGR emerges, often entering the role with only a passing understanding of the UK academic environment. The model of the Ph.D, its length, assessment, and structure of training provided, varies across international and institutional contexts (Louw and Muller, 2014). Expectations can also vary within institutions, between departments with different regulations and divergent disciplinary traditions (Phillips, 2010). The Ph.D is largely seen as a singular endeavour, with the metaphor of a journey frequently invoked within the education literature, an apprentice in the process of becoming, finding their own voice as a researcher (Batchelor and Napoli, 2006; Lynch and Kuntz, 2019). The end goal of this journey varies, and Phillips (2010) suggests a division between supervisors, primarily in the humanities and social sciences, who see the desirable output of an autonomous researcher, whereas some, primarily in the natural sciences, see it as a training process to produce research assistants. The prospective candidate must therefore negotiate the often-divergent aims and expectations of themselves, their supervisors, funders, university, and examiners.

In recent years, the model of the interdisciplinary Doctoral Training Programme has become more common (Doonan et al., 2018). Various studies exist investigating how contentions along disciplinary lines shape these PGRs, through the fostering of idea flow and creativity, but also inducing personal insecurities relating to depth of knowledge and future job opportunities (Haider et al., 2018; Knaggård et al., 2018; Mountford et al., 2020; O’Meara and Culpepper, 2020). Such programmes vary as to what support/training is provided (Killion et al., 2018), and individual Ph.D projects often end up being interdisciplinary in name only (Lindvig and Hillersdal, 2019). Villeneuve et al. (2020) present a collective autoethnography documenting the experiences of eight PGRs from various disciplinary backgrounds within the same research group. They present the shared Ph.D journey in this context as an ongoing process of developing a mutual understanding of each other and developing a higher level of intersubjectivity. In the absence of an overarching shared research project, the authors question the extent to which their group was a team. Despite sharing space, and co-organising events, each Ph.D remained an individual project. Nonetheless, the space sharpened critical and political thinking, and fostered negotiations towards a common language. Such findings have been echoed elsewhere, with the development of more reflexive scholars argued to be a key result of such shared experiences (Cuevas-Garcia, 2015; Haider et al., 2018; Knaggård et al., 2018).

Such reflexivity engenders a greater consideration of identity for interdisciplinary scholars through reflection on how their practice fits within the wider academic landscape (Felt et al., 2013; Holley, 2015; Lynch and Kuntz, 2019). Here, the PGR must negotiate what Dooling et al. (2017: 576) describe as ‘dual loyalties’: expected to fit the disciplinary mould of their home department, while simultaneously being an interdisciplinary scholar. Abdicating disciplinary identity can lead to feelings of being an outsider or ‘intellectually homeless’ (Balaban, 2018). On the contrary, Lindvig (2018) suggests that returning to the safety and protection of the home discipline to write leads to a suppression of creativity and experimentation. Cuevas-Garcia (2015) therefore argues that the interdisciplinary researcher must carefully navigate conflicting constructions of the value and rigour of interdisciplinary work to be able to present themselves as a serious researcher, thereby satisfying the institutional regulations demanded of them. Such a balancing act is personally demanding on the student, with a steep learning curve, and overload of competing expectations, often leading to burnout, anxiety, and feelings of inadequacy (Andrews et al., 2020; Balaban, 2018; Haider et al., 2018; Stanley, 2015).

Interdisciplinary PGRs’ anxieties are particularly referenced in relation to future employment within the sector. The challenges faced have been conceptualised in terms of the challenge in carving out an individual research profile (Felt et al., 2013), marketing and repositioning oneself for different audiences (Cuevas-Garcia, 2018; Holley, 2018), and insecurities about the depth of knowledge in relevant areas (Haider et al., 2018). Despite these perceptions, there seems to be a lack of empirical evidence to suggest interdisciplinary PGRs find it more difficult to obtain further employment (Holley, 2018). Various analyses suggest that pursuing an interdisciplinary Ph.D does not appear to hinder timely completion, or obtaining jobs of choosing (Carney et al., 2011), and that interdisciplinary graduates are more likely to be in academic careers than their monodisciplinary counterparts (Millar, 2013). A longitudinal study of 9 interdisciplinary Ph.D graduates by Holley (2018) revealed that the actual barriers and anxieties faced by the participants relate to the sector itself being hostile to ECAs, such as the time-intensive nature of post-doctoral research, the insecurity of short-term contracts, lack of support relating to maternity and childcare, personal sacrifices relating to work-life balance, and the frequent need to relocate.

Less emphasis has been given to the lived experiences of individual researchers engaged in interdisciplinary collaboration (Callard et al., 2015; Hillersdal et al., 2020; Stanley, 2015). Where this has occurred, focus tends to be on reflection of the challenges and successes of interdisciplinary collaboration along disciplinary divides (Freeth and Caniglia, 2020). Here Cuevas-Garcia (2018) has observed challenges faced by researchers whose discipline takes a minority position within a project, citing the recurrent need to assert themselves and justify the validity of their methods. Freeth and Caniglia (2020) challenge assumptions that researchers entering interdisciplinary collaborations already possess the necessary skills to work effectively together. They emphasise that such collaboration not only involves the confluence of disciplinary identities, but interpersonal dimensions such as divergent “beliefs and worldviews, normative and political orientations, embodied life experiences, and personal dispositions” (2020: 24). Other authors have also observed that the time required to learn how to collaborate in such a setting is greatly underestimated, thus leading to a need to revise the ambitiousness of the original research goals (Fam et al., 2020; Villeneuve et al., 2020). The role of the PGR within collaborative interdisciplinary work has been framed in terms of three dichotomies by Fam et al. (2020); the tension between the goals of the wider project and the expectation for students to finish their individual projects in time, the struggle to balance interdisciplinary outputs with the disciplinary standards of their home departments, and the need to learn across diverse knowledge bodies and methodologies while producing research outputs. Through these tensions, the collaborative success seemingly lies in direct competition with the goal of obtaining a Ph.D (Killion et al., 2018).

## Methodology & context

This study centres on and interrogates our own experiences as interdisciplinary PGRs, both within our specific personal contexts, the impact of conducting our Ph.Ds as members of a large interdisciplinary project, and within the wider political context of the contemporary British university as set out in section ‘The place of the interdisciplinary PGR within the British University’. The above literature illustrates the landscape in which we, the authors, entered the British academy as PGRs, stepping into an interdisciplinary research group formed by a single large project grant (2015–2020). This was located at a Russell Group^1^ university, corresponding to Sousa’s (2011) description of a ‘Mode-2 institution’.

### The Project

The Project onto which we were recruited had the central aim to take a holistic approach to understanding sustainable cities by using an explicitly ‘interdisciplinary’ project team and their skills. The Project team was composed from a range of disciplines, including sociology, economics, geography, physics, engineering, mathematics and computer science. The work was initially divided into six cross-cutting themes: Environmental; Social & Cultural; Economic; Measurement & Data; Modelling & Optimisation; and Policy & Governance. Owing to the institutional context of a large research intensive university, the Project occupied an uncertain space as an independent unit, with each of the participants affiliated or registered within a disciplinary department.

We developed our Ph.D theses as PGRs institutionally registered within geography: Phil (he/him), physics: Ben (he/they), and sociology departments: Ashley (she/her) and Hannah she/her), but were physically based within a shared office, which sat apart from our departmental spaces. We were originally hired to individual Project themes (Ben, Hannah, Phil), and as a project ethnographer overseeing all themes (Ashley). Our institutional disciplines corresponded to our previous degrees, except for the two sociologists; previously, Ashley studied international relations and Hannah political and administrative science.

The Project’s research group included 24 researchers: eight senior academics, six post-doctoral research fellows, and ten PGRs, with a roughly equal split between the social sciences (sociology, geography, economics) and natural sciences (physics, computer science, mathematics, engineering). Originally, researchers were hired to respective themes within the project, however as the project matured the boundaries of these themes became increasingly blurred and new informal themes formed. We contributed to the wider project in terms of regular “update and integration” meetings, workshops, seminars, both formal and informal discussion of potential ways to collaborate, and subsequent pursuit of interdisciplinary ‘micro’ projects. Meetings mostly took place within the building where the PGRs and postdocs were co-located, within a 5–10 min walk from our institutional disciplinary homes. While being in and around a shared space often felt like a privilege, some of us (Ashley, Hannah, Phil) began to attempt to bridge the distance—physically, relationally, and professionally—to our disciplines by attending their shared offices for one or two days a week.

While the Project was conceived as interdisciplinary, it was situated within the largely disciplinary structures of a British research university. Consequently, disciplinary norms of doing research and being recognised as a researcher applied to everyone in the Project and were—in our experience—rigidly imposed at the Ph.D level. Examples include assessment procedures and the need to submit progress reports and annual reviews within our disciplinary homes. This produced a tension between the disciplinary conventions demanded by departmental regulations and expectations and attempts to deviate from these norms through interdisciplinary work.

### Methodology

Similar to Trussell et al. (2017) and Christensen et al. (2021), we use personal reflections to interrogate our experiences, through an approach that is coherent with Tripathi et al. (2022)’s ‘retrospective collaborative autoethnography’ (RCA). We use Brookfield’s (2009) conceptualisation of ‘critical reflection’ to analyse and contextualise our ‘data’. Tripathi et al. (2022) outline RCA as a “method of enquiry [that] involves researchers interrogating their auto-biographies and observations retrospectively” in a collective and collaborative manner (p2). The authors argue that a collaborative approach confronts some of the limitations of standard autoethnographic research in providing transparency, accountability, & trustworthiness, as well as an enhanced opportunity for reflexivity. Further, it is argued by Tripathi et al. that the retrospective approach, i.e., the research process occurring after the described events have taken place, makes the analysis freer from bias due to the researchers being able to take an outside perspective.

Brookfield’s (2009) conceptualisation of ‘critical reflection’ “calls into question the power relationships that allow, or promote, one particular set of practices over others” (p294). Within our analysis, we therefore focus on the inherent power dynamics encapsulated within our observed themes, drawing out their wider structures and consequences. Accordingly, “[s]omeone engaged in critical reflection always asks whose interests are served by particular codes of practice, and stays alert to the way they are embracing ideas and behaviours that are subtly harming them” (Brookfield, 2009: p. 294). With critical reflection being a contested idea and not an unequivocal concept (Brookfield, 2009: p. 296), we do not solely subscribe to one of the intellectual traditions informing its use. Instead, we provide insights in relation to two purposes of critical reflection within our specified context: (1) to investigate power relationships and (2) to uncover hegemonic assumptions (Brookfield, 2009: p. 301).

The primary data this study relies on was collected informally and unsystematically over the course of our everyday experiences on the Project in the form of research notes, and memory. Through discursive and reflective techniques this experiential data was collated, compared, and analysed, resulting in the contribution that this paper makes. This work has come together over the course of around 3 years, both during and subsequent to our submission and examination of our respective Ph.D theses. Over the course of the Project we, the authors, regularly exchanged our views about the collaboration and our individual Ph.D research, sharing our fears, hopes, frustrations, and progress. We consequently became a support group, and our friendship continues having all left the city to which we had moved for the Project. Feeling that collectively our experiences were valuable not only to other researchers in similar positions, but as a contribution to the academic literature, we began to discuss our thoughts more formally as the evolving concept for this paper came together.

Our informal discussion thus began to transform into something more formal, and we began to have semi-structured meetings in which we gradually massaged our reflections into a theoretical framework. We began several working documents onto which key discussion points from our meetings were transcribed, and then subsequently developed by each of us in turn ‘offline’. Thus, at several points we translated the data of our individual experiential thoughts relating to a number of pertinent topics into written reflections, and vignettes, which were then discussed as a group and added to with complementary and contradictory reflections from each of the other authors. By adopting Brookfield’s (2009) framework of critical reflection to analyse our data, we used this iterative process to extract several common themes, which we describe and interpret in the subsequent sections: asymmetries of risk, of disciplines, and of knowledge. Our reflections are therefore retrospective and thus informed by our individual experiences following the completion of our Ph.Ds.

### Limitations

As an autoethnographic approach, our work comes with the critical challenges already outlined by many authors (see e.g., Forber-Pratt, 2015; Lapadat, 2017). Trapathi et al. (2022) characterise challenges relating to collective and retrospective autoethnography along the dimensions of individual bias; selective analysis; change of participant attitude; logistics; situational limitations; and influencing observations of other authors. They argue a combination of retrospective and collaborative approaches lowers many of these risks, through, e.g., the use of multiple voices to minimise individual biases, and the space put between the subject by the retrospectivity. The legitimacy granted to autoethnography as method has also been questioned in various places (Wall, 2008), we believe our reflexivity in relation to outlined challenges legitimates our approach.

We employ *the Project* as our corresponding case study. When reflecting on this single case of an interdisciplinary research project, one may ask how far—if at all—our conclusions are generalisable. Indeed, the claimed impossibility to generalise from a small n is a major objection against the case study method. In response to this critique, we choose to follow Flyvbjerg’s emphasis of “the force of example” (2006: p. 228). He puts formal generalisation into perspective by identifying it as “only one of many ways by which people gain and accumulate knowledge” (Flyvbjerg 2006, pp. 226–227). Thus, our exemplar should provide concrete and context-dependent insights in relation to understanding interdisciplinarity, dynamics of disciplines, and the position of the PGR.

Our situatedness necessarily impacts our experiences, views and potential biases; we partly occupy an intersection of positions, privileged or otherwise, and most notably arrived at the opportunity to pursue a funded Ph.D at a Russell Group University. Our positionality therefore brings ethical implications, particularly for Ashley, who was the ethnographer of the Project. Although we all bring with us our reflections from the project environment, Ashley’s reflections stem from her experience collecting data about the participants on the Project, which include the other authors of this paper. However, any reflections about other Project participants are left behind, to maintain ethical integrity and focus of this research paper. This work draws solely on the experiences of the authors, and does not attempt to speak for other members and PGRs engaged with Project. The focus of this work is not an evaluation of the Project’s success, nor a commentary on individual personalities or ways of working, but an investigation of the wider dynamics at play within the context in which we undertook our Ph.Ds.

While we frame our discussion around the ‘British context’ it is necessary to note how our positionality has shaped our perception of this, and allay any concerns about universalism. Certainly the historical and political context has shaped something broader than our own experiences, and the ‘British academy’ has been written about extensively. It must be emphasised, however, that the hierarchical structure of the academic context, principally but not solely captured in the distinction between ‘pre-’ and ‘post-92’ institutions (with the former category, which includes the ‘Russell Group’, being research intensive institutions), means that there is no truly homogenous British academic context. Nevertheless, despite the uneven distribution of impacts across these institutions, it should be noted that the issues outlined in section ‘The place of the interdisciplinary PGR within the British University’ impact all British universities.

Of final note is the impact of the interpersonal and professional relationships that we developed both with other members of the Project and with each other. Tucker et al. (2016) present friendship among co-authors as a prominent factor influencing the selection of co-authors and collaboration. While this allowed us to discuss with each other openly in the development of this work, without holding back disagreements or criticism, it is nevertheless an important factor to note as a potential limitation and the need for greater reflexivity here.

## Critical reflections of our contextual experience

In what follows, we build upon our formal discussions and written reflections of our personal journeys as PGRs within the context of the interdisciplinary Project. Through an iterative process, we synthesised our experiences under three themes relating to ‘asymmetries’ or power differentials, which we have observed and experienced, and the hegemonic assumptions at play. In doing so, we reflect upon how the interdisciplinary nature of the Project impacted our experiences of pursuing a Ph.D.

### Asymmetry of risk

As we have outlined, interdisciplinary research is often considered to be riskier than traditional disciplinary work (Byrne, 2014; Cuevas-Garcia, 2015). The Leverhulme Trust, one of the most prominent UK funders of interdisciplinary research emphasises its “reputation for encouraging higher-risk research, which is often therefore fundamental or curiosity-driven—so-called ‘blue skies’—and pan-disciplinary” (Leverhulme Trust, 2021). Where the burden of this higher-risk lies, is important to understand and is the focus of this section.

As highlighted in section ‘The rise of interdisciplinary research’, any interdisciplinary research requires an initial period of explanation, negotiation, and agreement to align the many methods of knowledge production being incorporated, as well as the specific disciplinary practices (Gieryn, 1983). If not appropriately factored into the original project proposal, the additional time required to do this “boundary work” puts the timeline of any interdisciplinary project at risk, creating subsequent risk to all participants. From the PGR’s perspective, their increased precarity—due notably to the finite time in which they are required to complete their thesis, and for which they may receive their stipend to enable them to do so—means that this subsequent risk falls unevenly on their shoulders.

This time pressure was observed within the Project. At the outset, and with a seemingly infinite amount of time available to explore interdisciplinary avenues, the risk of the boundary work did not appear too great despite more time than expected being taken up in meetings, and ad hoc attempts to stimulate interdisciplinary activity. The PGRs were happy to engage in these activities and contribute to side activities in line with the project aims, in addition to the core work building towards the ultimate completion of their own theses. However, as the Project proceeded and the many Ph.D milestones and deadlines came and went, necessity dictated that anything ancillary to completion of the thesis was abandoned, or at best postponed to an unspecified time post-submission. Aligned to this risk was a creep back towards each researcher’s ‘home discipline’ as the thesis submission also brought with it anxiety over what came next. This anxiety included the immediate concern over what would replace the postgraduate stipend as the primary means of income while the thesis was finished, but also what research papers or career opportunities could be developed following receipt of the Ph.D. None of us submitted within the respective windows for which we received stipend payments. To fill this funding gap, after varying lengths of living off personal financial savings, three of the researchers secured part-time employment within the sector (Ben, Hannah, Phil), while the other won a postgraduate fellowship (Ashley).

As has been shown, postgraduate researchers face pressures to submit both their thesis and relevant journal papers (Cairns et al., 2020) to enable progression in their academic careers. Within the Project our production of journal papers was undertaken both during, and after the completion of our theses, with the majority submitting to leading journals in their home discipline (Ashley, Hannah, Phil). Journal citations are the currency of ECAs, used to secure relevant post-doctoral work, which is in turn used to win research grants, and secure less precarious employment. These latter two stages increasingly require interdisciplinary experience, but the first, acceptance of papers in journals, is still very disciplinary bound. Many of the high-ranking journals span a less diverse set of disciplines than their lower ranked counterparts (Rafols et al., 2012), and still focus on long-standing disciplinary debates, seeking papers that offer answers to related core questions. In taking the time to navigate the boundaries of the many disciplines pursuant to a successful interdisciplinary project, the depth achievable by a postgraduate researcher in any one discipline will inevitably have to be sacrificed, and according to Byrne (2014), “there’s a risk of ending up being *[perceived as]* an expert in nothing”. Certainly, the ability of the researcher to produce journal papers that speak loudly to the core questions in these high-ranking disciplinary journals is put at risk, reducing their chances of a successful outcome, and jeopardising their early career development.

This is not to say that senior researchers and members of such a project are without significant risk themselves. The reputational risk of a failed or poorly delivered project poses a threat to career development at any stage. However, the risk for PGRs is more tangible—failure to submit a thesis or to be awarded a Ph.D, and no accepted publications—and can prevent entry to the academy in the first place. For senior academics the deliverables can be as minimal as a brief annual report, in which the failure of a project can be characterised as learning and part of the experimental nature of this inherently riskier research. The institution will still demand publications from the project, but data gathered during the process of any such failure can still be used to populate papers, complemented and supported by an already strong disciplinary knowledge enabling core disciplinary questions to be incorporated. For those in senior positions, their risk is also hedged as their time commitment on any single interdisciplinary project is usually low, sometimes as little as a few days a month, and they are likely to be involved in multiple interdisciplinary and disciplinary projects at any one time. Their exposure to reputational risk is therefore low and more manageable. For PGRs, the interdisciplinary project within which they are developing their Ph.D thesis is most likely their only project, or certainly it will account for most of their research. For them, very little is hedged, their proverbial eggs are in one interdisciplinary basket.

### Asymmetry of disciplines

The asymmetry experienced between the natural and social sciences in both allocation of resources and representation was experienced on the microlevel within the Project, where practices reinforcing the hierarchy of the disciplines (Cole, 1983; Gardner, 2013) could be felt. This hierarchy tends to put the natural sciences at the ‘top’, because positivist knowledge is privileged, and it is assumed that the natural sciences have high levels of consensus and uniform approaches in their theory, methods, and problem significance (Cole, 1983). Social scientists encountering natural scientists on the Project partly felt that their contributions as research professionals were undermined due to a misunderstanding of the role of their disciplinary expertise. In addition to hierarchical structures in the academy, natural scientists and social scientists differed in the cultural practices present in their respective disciplines in their ethical norms, authorship standards and Ph.D requirements. While these practices could go on as unnoticed tacit knowledge within a monodisciplinary setting, these differences were highlighted when diverse disciplinary cultures met on an interdisciplinary research project.

While the Project was interdisciplinary in nature, it was headed by principal investigators from the natural sciences, which had clear implications. The leadership imagined that simulation models would compose the central output of the Project as well as the principal site of interdisciplinary knowledge integration. Therefore, the social science and public policy researchers were expected to support the research aims by publicising the models to policy stakeholders and provide quantitative ‘model inputs’ to the natural scientists. From the perspective of the social scientists, interdisciplinary configuration often resembled a ‘support-service’ mode (Barry et al., 2008). While interdisciplinary project configurations do not necessarily need to resemble an ‘integrative-synthesis’ mode, favouring more symmetrical contributions from disciplinarians (Barry et al., 2008), the conflict lay mostly in the expectations from the respective researchers about what was considered a valuable contribution. While natural scientists on the Project were seeking help in supporting their existing model construction through indicators or communication with stakeholders, they viewed this as a legitimate form of collaboration. However, the social scientists on the Project did not perceive this collaboration as a valuable use of their research skills. This was reinforced by their own perceptions and the requirements outlined by the disciplinary schools.

In Ashley’s experience, her role was initially outlined as an ethnographer of the interdisciplinary process, acting as a collaborative facilitator, and seeking the ingredients needed to facilitate successful interdisciplinary collaboration. However, when she presented this research project to her home discipline, the work was not deemed critical enough to merit a sociology project at the doctoral level. What was considered a legitimate contribution and service to the interdisciplinary Project, was not considered ‘good science’ by her disciplinary home. In another example, social science PGRs on the project were approached by modellers to provide the ‘most important’ factors from their research domains to act as input to help build a simulation model. From the perspective of the modeller, this request was an attempt at collaborating with the social scientists, however from the perspective of the social scientists, this request was considered a ‘service’ and not a valuable contribution that utilised their critical skills. In these two examples, the social scientists were seeking critical research approaches, and they did not feel that the collaborative *‘*subordination-service’ configuration of the Project provided them the framework to do so.

The social scientists did not adopt the natural scientists’ ways of working on the modelling and preferred to address their own research aims that would award them a Ph.D. In addition, their disciplinary backgrounds encouraged them to be critical of modelling approaches, rather than adopting them without question. There is a danger of social scientists being co-opted by positivist methods in interdisciplinary collaborations because of a lack of true knowledge integration and exchange. This co-option causes some social scientists to approach interdisciplinary projects with caution (Holmwood, 2010). Owing to sociology’s wide breadth of application and broad definition, social scientists are more likely to have their roles seen as science communicators (Robinson et al., 2019), specialists in ethical and social implications (Calvert and Martin, 2009), research carers (Viseu, 2015), and even recruiters (Goulden et al., 2017).

The experience of needing to choose between doing interdisciplinary collaboration or work that would merit a Ph.D was different for Ben as a natural scientist. Encountering issues in applying a positivist methodology to social phenomena (Purvis, 2020), Ben was able to exploit the relatively long leash allowed by his home department’s doctoral regulations to shape his research goals by employing methodological aspects from the social sciences. Although his methods were deemed ‘non-traditional’, and the divergence of expectations between natural and social scientists led to lengthy thesis revisions after examination, Ben still earned a Ph.D in the natural sciences.

The divergence between the natural and social sciences is not limited to epistemological or methodological differences but extends to their disciplinary cultures as well. Authorship practices vary across the natural and social sciences, creating additional challenges for interdisciplinary manuscript development (Oliver et al., 2018). Divergent norms are tacitly accepted within respective disciplines, and often assumed as a universal practice if not confronted with a different way of doing things. A joint paper drafted by PGRs from the natural and social sciences brought to light these different disciplinary cultures. Senior academics from the natural sciences assumed that they would be named on the paper, as is the common and accepted practice in their discipline. This assumption was met with surprise and rejected by the social science authors of the paper. This practice from their perspective was controversial, as a significant contribution was not made by senior colleagues and adding more authors could dilute the perceived contribution of the social scientists, who needed strong publications to start their career in academia. This conflict of authorship norms disproportionately impacted the social scientists here.

The cultural practices, methodological approaches and departmental requirements are all examples of boundary work taking place at the disciplinary level (Becher, 1989; Gieryn, 1983). While navigating the Ph.D thesis, PGRs encountered preferred norms of data collection and thesis structure. For example, Hannah’s first year review of her work resulted in senior academics suggesting that she spent more time in the social sciences office, rather than the shared interdisciplinary Project space. The rationale was that her work needed more influence from traditional social science thinking, and informal exchange as well as physical exposure to other social scientists would give her that. Indeed, following this advice proved effective to better adopt the norms of sociological Ph.D research.

Different disciplinary cultures conflicted with one another, but also the interdisciplinary aspirations of the Project. Hierarchies were experienced between the natural and social sciences as well as between doing disciplinary work and interdisciplinary collaboration. Disciplinary contribution was a priority for social sciences particularly, where their departments exerted more pressure to conform to a ‘social science Ph.D’. Conflict in research is not necessarily a bad thing, however it does require extra energy, time, and emotional labour to address assertively. Given the individual career priorities and the respective Ph.D requirements for junior researchers, it meant that addressing this conflict became a secondary objective on this fixed-term project.

### Asymmetry of knowledge

Throughout the Project, we observed that we and other junior colleagues had less knowledge and experience in relation to research compared to more senior colleagues; and we felt less confident to challenge them and ways of ‘doing’ interdisciplinarity. Uncertainties were present throughout the hierarchies within the Project but were particularly salient for PGRs who pursued explicitly interdisciplinary thesis projects.

A significant example for these uncertainties is how Ben experienced and negotiated their interdisciplinary Ph.D trajectory, with a recurrent fear that their divergence from disciplinary conventions left their work open to critique from senior researchers. While they had grappled with a wide breadth of concepts and approaches within their thesis, they were acutely aware of areas where this breadth had been accompanied by a lack of depth. Since they were writing for examiners from a different disciplinary background to themself and their supervisors, Ben was worried about divergent expectations for their thesis. Uncertainties about interdisciplinary research thus had real consequences: ultimately Ben had to make significant corrections to their thesis due to failing to satisfy expected disciplinary norms from one of their examiners. While more senior colleagues may experience similar uncertainties, their professional validation does not usually depend on dealing with them to a similar extent.

The tension between the breadth and depth of knowledge was a recurrent theme for all of us. With ultimately limited time to familiarise ourselves with academic literatures and approaches, the Project necessitated developing an appreciative breadth. In contrast, fellow discipline-based PGRs outside the Project often focused on in-depth disciplinary knowledge. In this context, situations arose where we perceived our own disciplinary knowledge as insufficient or where we interpreted reactions of others indicating this. This included feeling the need to keep a view on an ever-increasing list of journals, while disciplinary colleagues are perceived to become masters of a few core journals, being able to recall historical debates, which the interdisciplinarian is unaware of. This led to feelings of inadequacy for the interdisciplinary PGRs, and feelings of anxiety, particularly in the lead-up to examination.

In the wider context, there is the perception that high impact journals and prestigious conferences are based on disciplinary knowledge and debates. In line with many academic valuation standards, disciplinary knowledge production is often more prestigious than its interdisciplinary counterparts. At times, we felt that the breadth of knowledge we gained within the Project partially hampered a more expert disciplinary knowledge, leaving us disadvantaged in relation to the disciplinary performance criteria and fostering an accompanying lock-in effect. Prioritising interdisciplinary over disciplinary knowledge—with the latter being more hegemonic within academia—might therefore reduce one’s chances at an academic career. We argue that this is particularly the case at the beginning of a career compared to senior colleagues who are likely more established in their disciplines. Our experiences affirm how interdisciplinary research can come with feelings of inadequacy in relation to competing expectations (Andrews et al., 2020; Balaban, 2018; Haider et al., 2018; Stanley, 2015), as explored in section ‘The rise of interdisciplinary research’.

Closely related to the boundary work discussed in the previous, there exists an asymmetry of knowledge in identifying disciplinary boundaries and norms. This encompasses insecurities around knowing one’s discipline, its corpus of literature and demands of qualifying oneself for a Ph.D within it. This manifested in a context of boundaries between disciplines appearing as fluid, in flux, tacit and contested. Paradoxically, these boundaries and norms were sometimes rigidly enforced as described already, though sometimes blurry and implicit. For example, a natural scientist in the Project suggested that Ashley use recording equipment to capture candid office conversations that could be the starting points of collaborative opportunities. Senior social scientists in Ashley’s home department were alarmed at this suggestion due to research ethics. As a PGR unaware of how different the perception of ethical norms would be, Ashley had to navigate the best way forward, between contributing to the interdisciplinary Project, and adhering to the norms of her home discipline.

Knowledge of norms about what is or is not the social scientist’s way of doing things felt like a trial-and-error process. As junior researchers, PGRs come up against these norms accidentally, because they are exposed to alternative disciplinary practices and lack the experience to acquire the tacit knowledge of their home discipline. The social science department in this case imposed their acceptable disciplinary boundaries and had the power to enforce them, as ultimately the department would award a Ph.D at the end of the research. Such issues of (not) knowing disciplinary boundaries—or even not being able to know, given their contested and implicit nature—appeared more critical for those working towards Ph.Ds than for those having passed this threshold.

Our accounts here particularly resonate with the insecurities and anxieties previously described within the context of the contemporary neoliberal academy. We have shown that hierarchical power relations and gatekeeping are important reasons why a limited understanding of interdisciplinarity is more problematic for junior than for senior researchers. Academic reward systems often favour those who already have advantages, e.g., in terms of publications, research methods, prestigious institutions or funding. ‘Losers’ in these systems are those who are less known, without institutional affiliation or scientific offspring—and can therefore be underrated in relation to their actual performance (Bol et al., 2018). Our reflections demonstrate how ways of doing and knowing interdisciplinarity are strongly influenced by hegemonic assumptions that disciplines and more senior researchers continue to define through disciplinary norms. In this context, requirements and calls to work interdisciplinarily can complicate career progression, particularly for junior researchers who are expected to fulfil existing disciplinary requirements—but may not even know about these diverging interests when starting a Ph.D. Our research therefore supports suggestions by Fam et al. (2020) and Killion et al. (2018) who describe similar antagonisms between interdisciplinary research and goals of a disciplinary Ph.D (cf. section ‘Navigating this space as a PGR’ above).

## Discussion

Through our critical reflection of our experiences as PGRs undertaking Ph.Ds as part of a collaborative interdisciplinary project, we sought to understand in which ways and to what extent the interdisciplinary nature of this environment shaped us as researchers in our formative years. Contrary to suggestions within the literature (Byrne, 2014; Cuevas-Garcia, 2015; Trussell et al., 2017), we found that our own experience of uncertainty and precarity as junior researchers stemmed principally from pre-existing structures within British academia, rather than being imparted through the interdisciplinary nature of the project in which we were involved. We thus argue that this environment presents itself as a wide-angle lens for examining existing asymmetries within the neoliberal British university. Through iterative reflection, we characterised pre-existing tensions within the academy as ‘asymmetries’, reflecting uneven power relationships along the dimensions of risk, disciplinary hierarchy, and knowledge.

### Three asymmetries

First, we outlined what we have referred to as the *asymmetry of risk* within the neoliberal academy: those with power can take greater risks. This is evidenced within the wider research system by the concentration of funding in large grants for the creation of hierarchical ‘research teams’, rather than in lots of smaller ones for autonomous researchers (Aagaard et al., 2020). Aside from biases towards larger research institutions, most funding opportunities greatly favour more senior academics with previous successes (Bol et al., 2018), and open-ended contracts offer greater security and flexibility to compete within the funding landscape. The ‘publish or perish’ imperative disproportionately impacts junior scholars without an established publication track record, with the first years in the sector often seen as crucial in the make-or-break of an academic career. Within the wider academy, this asymmetry of risk is also witnessed through the proliferation of casualised contracts, and sub-living wages, while senior managers engage in speculation with university finances. As PGRS, this asymmetry of risk manifested in fixed deadlines associated with funding, and financial penalties for exceeding the bounds of ‘timely completion’. The nature of the Ph.D stipend as unwaged income also places PGRs in the precarious position of being ineligible for various financial products. Additionally, the current metrics of ‘success’ within the academic sector, by leaning heavily on ‘tangible outputs’, impact, publications, public engagement, and co-production, side-lines the need for support and development of junior researchers. Yet the nature of the Ph.D, requiring a deliverable in the form of a thesis submitted in a ‘timely’ manner, affords the PGR little space to engage in more risky activities such as engagement and collaborative activities that do not form a direct part of their research design.

The *hierarchy of disciplines* forms an asymmetry in which the hegemonic power that currently sits with STEM subjects leads to marginalisation of social sciences, the arts, and humanities. This is currently playing out within the UK sector in the form of a sustained assault on marginalised subjects, both within the press and government rhetoric, and cuts and redundancies under the guise of post-pandemic prudence. This asymmetry reproduces current normative values within wider society and the ideological tilt of a government increasingly hostile towards these disciplines. The Project gave us a unique vantage point of the asymmetries of disciplines that exists today in academia, viewed in terms of micro interactions between researchers from divergent disciplinary backgrounds. This was evident in the makeup of the Project itself, with the existing hierarchy enforced through the primary leadership of the Project being STEM-oriented, with social scientists often imagined as providing a ‘support’ role. Additionally, in our experience, the social science PGRs were afforded a lot less flexibility in their research design and divergence from ‘disciplinary norms’ than our natural science counterparts.

Finally, we characterised an *asymmetry of knowledge*, in terms of the power afforded through experience. This may be exemplified by junior colleagues perceiving senior colleagues as more of an authority in terms of knowledge of research practice, norms, and theory. Within the wider academy, junior colleagues such as Postdocs and PGRs are often viewed as a source of cheap (or even free) labour. Indeed, in some fields, this may be driven by the perverse rewards of ‘academic success’ leading to a shrinking amount of time for senior academics to pursue research agendas themselves. The way in which PGRs are gatekept from the academy as ‘Ph.D candidates’ and must ‘defend’ their work to the satisfaction of established colleagues demonstrates the explicit nature of this asymmetry. PGRs are viewed as apprentices regardless of previous experience, publications, or achievements, and their ‘knowledge’ is deemed insufficient until successful examination defence. Through our experiences as interdisciplinary PGRs however, we experienced that the breadth vs depth dichotomy introduces alternate ways of knowing, allowing us to eclipse senior colleagues in terms of breadth in some areas and depth in others. This was evidenced by a perceived lack of knowledge by senior colleagues of disciplinary norms outside of their own, and our practice of constructing our own (inter)disciplinary identities.

### Interdisciplinarity and the transformation of the academy

As we have shown, the PGR reflections included here come during a period of transformation for British academia as the more instrumental nature of research is encouraged. The underlying reasons for this emphasis stem from long-held epistemological debates surrounding the different approaches to creating and applying knowledge, manifested in a shift from Mode-1 to Mode-2 research (Gibbons, 1994). This aligns well with the need to work across disciplines to solve today’s ‘wicked problems’, such as inequality and climate change. This coincides with a period of particular financial strain caused by austerity and successive governments hostile to the public university, and is now being exacerbated by the uncertainty brought by both Brexit and the fallout of the COVID-19 pandemic. Attempts to cope with this strain are manifested in wholesale cost-cutting measures in the form of staff reductions and casualisation, the closure of departments, and rising tuition fees (Munro and Heath, 2021; Rings, 2021).

Despite increased commitments to interdisciplinarity, reinforced through academic funding regimes, UK universities are still largely disciplinary-oriented, as are the measures of success—including both the REF and the most prestigious academic journals. Indeed, Christensen et al. (2021) argue from their own reflections that interdisciplinarity is undervalued by both the academy and wider funding structures. The reflections presented here demonstrate some of the difficulties faced by interdisciplinary PGRs when comparing themselves to discipline-based peers. We highlighted these as the asymmetry of disciplines, which includes the perceived disadvantages of being academic nomads, as opposed to bringing a deep, and collective, understanding of a discipline and its historic debates and topics. This immediate difference in type and breadth of knowledge, we suggest, puts the interdisciplinary PGR at a disadvantage when it comes to aligning with REF criteria and being able to offer relevant background to high ranking, disciplinary focussed journal articles. It has also been found to influence research project choices, with some researchers deciding that disciplinary research was a quicker route to progress within a disciplinary structured academy while interdisciplinary projects were considered riskier (Byrne, 2014; Cuevas-Garcia, 2015). These locked-in disciplinary administrative, measurement, and reward procedures need to be addressed if interdisciplinary research is truly valued.

These developments create considerable anxieties for the interdisciplinary PGR. Asked to commit to a deep-dive into a highly specialised area of research, they must also navigate this tumultuous wider context to find future opportunities, and often immediate sources of income to support the completion of their thesis. Many of these anxieties also exist for disciplinary PGRs, but if the transformation to delivering more, or even solely, interdisciplinary knowledge is the intended outcome, then British academia must address both the inconsistent structure and values across all its institutions. Although PGRs viscerally experience this precarity in the university system, it is not directly due to the interdisciplinary nature of projects, but rather, the rise of precarious employment alongside interdisciplinarity as a symptom of the neoliberal academy.

### Transforming the Ph.D

Based on the critical reflection of our ambiguous experiences, we argue that the period of Ph.D study should be marked by fairer and more caring conditions. Through our position as PGRs in the interdisciplinary Project, we may have gained a broader view of the academic system than disciplinary PGRs, having observed various disciplinary practices and their interplay. Being in a formative and relatively dependent position, our identity as researchers was arguably much more shaped by the Project than that of our more senior colleagues. Yet our experiences are ambiguous. Despite the challenges of being in a marginal position in an interdisciplinary environment, we benefited from this both through the lens it offered, and through the skills we were able to develop. Accordingly, we have all used our interdisciplinary experience as a selling point when applying for scholarships or jobs and have drawn on it to inform our research. This seemingly supports Holley’s (2018) suggestion that there is a lack of evidence that interdisciplinary PGRs find it more difficult to find future employment than their monodisciplinary counterparts. In these ways, an interdisciplinary context may be particularly helpful to prepare for work both inside and outside of the academy.

While some of the asymmetries we have outlined could be argued necessary through the nature of the Ph.D as a training ground, other training does not necessitate such marked power differentials, or come with similar future job insecurities or such lengthy qualification periods. Additionally, the existing asymmetries relate to the discussed mental health crisis in the academy (Evans et al., 2018; Levecque et al., 2017; Wray and Kinman, 2021). It is therefore less of an individual problem, but systemic. In this light, the rise in PGR numbers is problematic when it is not accompanied by funding, job opportunities and broader structures to support PGRs to develop post-Ph.D. The pyramid-like structure of the academy is often not fully comprehended by prospective ECAs until they have entered the system. Such dynamics exacerbate issues like shaming, unhealthy or abusive work relations and patterns, as well as impacting job markets. A deeper consideration of systemic issues in relation to the Ph.D journey harks to the concept of ‘development ecology’ and its emphasis on the interplay between societal, organisational, and individual dimensions (Christensen, 2016). This provides a potential alternate frame for exploring the Ph.D journey, alongside Christensen’s (2016) suggestion to more fully consider resilience within this context.

Through our critical reflections, and discussion relating to reframing success, we suggest that we ourselves are a core output of the Project, regardless of any publications, ‘impacts’, or ‘successful’ interdisciplinary collaboration. Accordingly, we argue that the Ph.D is undervalued, as it is uncounted as an output and the thesis is typically not considered to be a ‘publication’. This is evident for example in accounting exercises such as the REF where the PGR is not classified as an ‘independent researcher’. On the contrary, the development and training of a new generation of critical scholars should be considered a major and important output. While considering the trained PGR themself as an output implicitly buys into the metricised logic of contemporary academia, such a reframing shifts the focus towards the nurturing of an individual researcher who is valued for their skill and expertise rather than just another cog in the generation of research impact. In doing so lies the implicit call to more deeply consider the PGR’s place within wider organisational, and indeed societal structures.

Recentring the Ph.D around the element of training and skill development and valuing the creation of skilled researchers may help to address some of the issues we have identified. The time PGRs devote to this journey should be meaningful and marked by fairer and more caring conditions. To this aim, the Ph.D model in the UK should recognise PGRs as staff, grant full funding until completion, and encourage work-life-boundaries. Funding should require the supervisor to be formally trained in supporting the ECA pathway, and preferably to have engaged with boundary work to better understand associated challenges prior to recruiting PGRs in interdisciplinary contexts. Many other skills can be promoted during a Ph.D, which can be of value both in- and outside of the academy. With their challenges in mind, interdisciplinary environments have great potential to foster such skills as teamwork, flexibility, social skills, understanding of pluralism, varied experiences, and relations in often international contexts.

## Outlook

While the Project provided the richness of our everyday experience as interdisciplinarians in an academic environment, it remains a singular example. The Project, our reflections and our positions all take place within specific contexts including, the location within a Russell Group University, continued employment within research roles in the Global North, our personal interest strengthening the position of the Ph.D in the UK, and our COVID-19 tinted lens, which highlighted issues of funding collapse, redundancies, and enrolment crises. In any other context or time, our views and conclusions may have developed differently. While we ourselves are experiencing the precarious nature of academia, we also recognise and acknowledge our simultaneous positions of privilege to be able to pursue careers in this competitive field. Valuable additional insight from the voices of the less privileged PGRs who are unemployed, had to leave academia against their will or who are simply in other institutions that don’t allow time to voice such concerns should find more expression to add to the conversation.

Examining the everyday effects of the wider systemic change in universities would benefit from a broader lens that looks out onto the wider consequences of rising interdisciplinary funding for employment at universities and resultant power structures. Crucially, a future research agenda here could employ a broader methodological toolset to overcome some of the shortcomings of an autoethnographic approach, and the specificities of a singular case study. Particularly pertinent is the expansion of our evidence base to examine to what extent our reflections match the experiences of other PGRs across a broader range of disciplines and institutional contexts (i.e., outside of a Russell Group institution).

Research here could work towards understanding where interdisciplinary research centres fit into a departmentally organised university. Who are the researchers that are more likely to work in these centres (if there is a pattern at all)? What implications does employment at a centre like this have on wider careers? In the context of rising precarity, this research could dovetail to investigate how employment dynamics in the research sector compare to other forms of employment. For example, in other private sectors or graduate schemes, how long does a training period last and what follows its completion? What other responsibilities outside of the job description are new employees/PGRs expected to consider? This comparison can help to better understand the nature or ‘function’ of a PGR position. Is the Ph.D a position of a researcher in training that is expected to further their research and career in academia, or is it comparable to the unpaid intern market, where internship labour is used to fulfil company tasks at a cost-effective level? Does the Ph.D function to prepare a PGR for a specific career in a university department, or does it provide wider marketable skills for employment outside of academia as a researcher?

The above proposal to examine the Ph.D’s function may open up possibilities to clarify the purpose of the Ph.D within the UK academic sector, and to make this transparent from the start. One of the purposes of this paper was to communicate that the function of the PGR is not obvious, and we were given conflicting and diverse instructions on how to complete our work. Is a (funded) PGR there to contribute to the work of a centre/department, or are they there to meet the objectives of a specific project? If they are expected to satisfy both, how much can these two objectives align? Is reframing the Ph.D as an important output of such projects a positive move, or could it lead to perverse consequences under the existing metric focused paradigm? Is the Ph.D an exercise in academic writing for future journal publications or is it more an end in and of itself (a rite of passage?) that stands apart from the day to day of an academic researcher? Creating a more transparent and consistent narrative about what is entailed in the Ph.D and what it can realistically offer a prospective student can help interested candidates make informed decisions about what responsibilities they take on before and after they embark on a Ph.D programme.

Our analysis has limited itself primarily to the level of the academy, only implicitly acknowledging transformations at the societal scale that have led to the construction of the neoliberal academy. Consideration of the interactions between these two levels are crucial, however, for dismantling the neoliberal academy in favour of a structure that prioritises care. The British academy is after all at the mercy of pernicious government policy, which includes the pervading neoliberal logic as well as the impacts of austerity. At the time of writing, the UK’s University and College Union is balloting its members for strike action, against the backdrop of a ‘cost of living crisis’, joining a nationwide eruption of trade union activity at a scale not witnessed in the UK since the 1970s.

At the outset, this paper sought to question how and to what extent the interdisciplinary nature of the Project, within which we pursued our Ph.Ds, shaped us as early-stage researchers. The findings from our critical reflections on this matter coalesced into themes, which we categorised in terms of asymmetries relating to risk, disciplinary hierarchy, and knowledge. Yet with reference to the literature on the neoliberal academy, interdisciplinarity, and the Ph.D experience, we argue that the interdisciplinary nature of the project acted as a lens through which we were able to more clearly observe and examine the asymmetries within contemporary academia. While we felt that ultimately we all benefited from the interdisciplinary nature of the project, these wider asymmetries were not conducive to a positive PGR experience. We believe then that questioning and reframing the role of the PGR is a necessary step in facilitating a much-needed transition to a university system, which promotes care for the individual and values personal success at all levels.

## Data Availability

Data sharing not applicable to this article as no datasets were generated or analysed during the current study.

## Footnotes

1 A grouping of 24 ‘elite’ universities, which while consisting of only 15% of higher education institutions in the UK, receive over 75% of research grant income (Russell Group, 2016).

## References

[CR1] Aagaard K, Kladakis A, Nielsen MW (2020). Concentration or dispersal of research funding?. Quant Sci Studi.

[CR2] Aboelela SW, Larson E, Bakken S (2007). Defining interdisciplinary research: conclusions from a critical review of the literature. Health Serv Res.

[CR3] Adeluwoye D (2020) How neoliberal reforms led to the student covid crisis. Tribune. https://tribunemag.co.uk/2020/10/how-neoliberal-reforms-led-to-the-student-covid-crisis. Accessed 04 Jan 2023

[CR4] Ahlburg DA (2020). Covid-19 and UK universities. Polit Q.

[CR5] Albert M, Laberge S, Hodges BD (2009) Boundary-work in the health research field: biomedical and clinician scientists’ perceptions of social science research. Minerva 47(2):171–194

[CR6] Andrews EJ, Harper S, Cashion T, et al. (2020) Supporting early career researchers: insights from interdisciplinary marine scientists. ICES Journal of Marine Science 77(2):476–485

[CR7] Balaban C (2018). Mobility as homelessness. Learn Teach.

[CR8] Barry A, Born G, Weszkalnys G (2008). Logics of interdisciplinarity. Econ Soc.

[CR9] Bartram B, Carruthers Thomas K, French A (2020). Queering the TEF. Challenging the teaching excellence framework.

[CR10] Batchelor D, Napoli RD (2006). Special issue: the doctoral journey: Perspectives. Educate.

[CR11] BBC (2020) More students say university not value for money. BBC News, 11 Jun

[CR12] Becher T (1989) Academic tribes and territories. Milton Keynes, Society for Research into Higher Education, England

[CR13] Bhambra GK, Gebrial D, Nişancıoğlu K (2018) Decolonising the University. Pluto Press

[CR14] Bol T, de Vaan M, van de Rijt A (2018). The Matthew effect in science funding. Proc Natl Acad Sci USA.

[CR15] Boliver V (2015). Are there distinctive clusters of higher and lower status universities in the UK?. Oxf Rev Educ.

[CR16] Brookfield S (2009). The concept of critical reflection: promises and contradictions. Eur J Soc Work.

[CR17] Brooks SD, Dean AS, Franklin-Phipps A (2020). Becoming-academic in the neoliberal academy: a collective biography. Gend Educ.

[CR18] Brown T (2004) PhD students or employees, which way should the UK go? National Postgraduate Committee

[CR19] Burki TK (2020). COVID-19: consequences for higher education. Lancet Oncol.

[CR20] Byrne S (2014) Interdisciplinary research: why it’s seen as a risky route. The Guardian, 19 Feb

[CR21] Cairns R, Hielscher S, Light A (2020) Collaboration, creativity, conflict and chaos: doing interdisciplinary sustainability research. Sustain Sci. 15:1711–1721

[CR22] Callard F, Fitzgerald D (2015). Rethinking interdisciplinarity across the social sciences and neurosciences.

[CR23] Callard F, Fitzgerald D, Woods A (2015). Interdisciplinary collaboration in action: tracking the signal, tracing the noise. Palgrave Commun.

[CR24] Calvert J, Martin P (2009). The role of social scientists in synthetic biology. EMBO Rep.

[CR25] Carney J, Martinez A, Dreier J (2011). Evaluation of the National Science Foundation’s Integrative Graduate Education and Research Traineeship Program (IGERT): Follow-up study of IGERT graduates.

[CR26] Casadevall A, Fang FC (2014). Specialized science. Infect Immun.

[CR27] Castán Broto V, Gislason M, Ehlers M-H (2009). Practising interdisciplinarity in the interplay between disciplines: experiences of established researchers. Environ Sci Policy.

[CR28] Christensen J (2016). A critical reflection of Bronfenbrenner’s development ecology model. Probl Educ 21st Century.

[CR29] Christensen J, Ekelund N, Melin M, Widén P (2021). The beautiful risk of collaborative and interdisciplinary research. A challenging collaborative and critical approach toward sustainable learning processes in academic profession. Sustainability.

[CR30] Christie F (2017). The reporting of university league table employability rankings: a critical review. J Educ Work.

[CR31] Chubb J, Reed MS (2018). The politics of research impact: academic perceptions of the implications for research funding, motivation and quality. Br Politics.

[CR32] Cole S (1983). The hierarchy of the sciences?. Am J Sociol.

[CR33] Cooper G (2013). A disciplinary matter: critical sociology, academic governance and interdisciplinarity. Sociology.

[CR34] Cornell B (2020) PhD students and their careers

[CR35] Corona Contract (2020) We Cannot Pause in a Pandemic—Response to Rocha and Marris. New Socialist, 22 May

[CR36] Croxford L, Raffe D (2015). The iron law of hierarchy? Institutional differentiation in UK higher education. Stud High Educ.

[CR37] Cuevas-Garcia CA (2015). ‘I have never cared for particular disciplines”–negotiating an interdisciplinary self in biographical narrative’. Contemp Soc Sci.

[CR38] Cuevas-Garcia CA (2018). Understanding interdisciplinarity in its argumentative context: thought and rhetoric in the perception of academic practices. Interdiscip Sci Revi.

[CR39] Cundy S (2020) Manchester’s Student Revolt. Tribune, 13 Nov

[CR40] van Dalen HP, Henkens K (2012). Intended and unintended consequences of a publish-or-perish culture: a worldwide survey. J Am Soc Inform Sci Technol.

[CR41] Darbellay F (2015). Rethinking inter- and transdisciplinarity: Undisciplined knowledge and the emergence of a new thought style. Futures.

[CR42] Datta A (2018) Negotiating difference in an interdisciplinary collaboration. Working Paper. Overseas Development Institute

[CR43] Davé A, Hopkins M, Hutton J (2016). Landscape review of interdisciplinary research in the UK.

[CR44] Dickinson J (2020) UKRI publishes its review of support for PGRs during the pandemic. Wonkhe, 11

[CR45] Doharty N, Madriaga M, Joseph-Salisbury R (2021). The university went to ‘decolonise’ and all they brought back was lousy diversity double-speak! Critical race counter-stories from faculty of colour in ‘decolonial’ times. Educ Philos Theory.

[CR46] Dooling S, Graybill JK, Shandas V, Frodeman R, Thompson Klein J, Pacheco RCS (2017). Doctoral Student and Early Career Perspectives on Interdisciplinarity. The Oxford handbook of interdisciplinarity. 2nd edn.

[CR47] Doonan F, Taylor L, Branduardi P (2018). Innovative training networks: overview of the Marie Skłodowska-Curie PhD training model. FEMS Microbiol Lett.

[CR48] Engelen E, Fernandez R, Hendrikse R (2014). How finance penetrates its other: a cautionary tale on the financialization of a Dutch university. Antipode.

[CR49] Evans TM, Bira L, Gastelum JB (2018). Evidence for a mental health crisis in graduate education. Nat Biotechnol.

[CR50] Fam D, Clarke E, Freeth R (2020). Interdisciplinary and transdisciplinary research and practice: balancing expectations of the ‘old’ academy with the future model of universities as ‘problem solvers’. High Educ Q.

[CR51] Felt U, Igelsböck J, Schikowitz A (2013). Growing into what? The (un-)disciplined socialisation of early stage researchers in transdisciplinary research. High Educ.

[CR52] Findlay AM, McCollum D, Packwood H (2017). Marketization, marketing and the production of international student migration. Int Migr.

[CR53] Flyvbjerg B (2006). Five misunderstandings about case-study research. Qual Inq.

[CR54] Forber-Pratt AJ (2015). “You’re going to do what?” Challenges of autoethnography in the academy. Qual Inq.

[CR55] Frank AK (2017). What is the story with sustainability? A narrative analysis of diverse and contested understandings. J Environ Stud Sci.

[CR56] Freeth R, Caniglia G (2020). Learning to collaborate while collaborating: advancing interdisciplinary sustainability research. Sustain Sci.

[CR57] Gardner SK (2013). Paradigmatic differences, power, and status: a qualitative investigation of faculty in one interdisciplinary research collaboration on sustainability science. Sustain Sci.

[CR58] Gibbons M (1994). The new production of knowledge: the dynamics of science and research in contemporary societies.

[CR59] Gibson C, Stutchbury T, Ikutegbe V (2019). Challenge-led interdisciplinary research in practice: Program design, early career research, and a dialogic approach to building unlikely collaborations. Res Eval.

[CR60] Gieryn TF (1983). Boundary-work and the demarcation of science from non-science: strains and interests in professional ideologies of scientists. Am Sociol Rev.

[CR61] Goulden M, Greiffenhagen C, Crowcroft J (2017). Wild interdisciplinarity: ethnography and computer science. Int J Soc Res Methodol.

[CR62] Goyette S (2016). Interdisciplinarity helps solving real-world problems. Reg Environ Change.

[CR63] Haider LJ, Hentati-Sundberg J, Giusti M (2018). The undisciplinary journey: early-career perspectives in sustainability science. Sustain Sci.

[CR64] Harvey D (2005). A brief history of neoliberalism.

[CR65] Helgesson G, Eriksson S (2019). Authorship order. Learn Publ.

[CR66] Hillersdal L, Jespersen AP, Oxlund B (2020). Affect and effect in interdisciplinary research collaboration. Sci Technol Stud.

[CR67] Holbrook JB (2017). The future of the impact agenda depends on the revaluation of academic freedom. Palgrave Commun.

[CR68] Holley KA (2015). Doctoral education and the development of an interdisciplinary identity. Innov Educ Teach Int.

[CR69] Holley KA (2018). The longitudinal career experiences of interdisciplinary neuroscience PhD recipients. J High Educ.

[CR70] Holmwood J (2010). Sociology’s misfortune: disciplines, interdisciplinarity and the impact of audit culture. Br J Sociol.

[CR71] Ivancheva M, Lynch K, Keating K (2019). Precarity, gender and care in the neoliberal academy. Gend Work Organ.

[CR72] Kezar A, DePaola T, Scott DT (2019) The Gig Academy: mapping labor in the Neoliberal University. JHU Press

[CR73] Kidd IJ, Chubb J, Forstenzer J (2021). Epistemic corruption and the research impact agenda. Theory Res Educ.

[CR74] Killion AK, Sterle K, Bondank EN (2018). Preparing the next generation of sustainability scientists. Ecol Soc.

[CR75] Kınıkoğlu CN, Can A (2020) Negotiating the different degrees of precarity in the UK academia during the Covid-19 pandemic. Eur Soc 23(sup1):S817–S830

[CR76] Klein JT, Klein JT, Mitcham C, Frodeman R (2010). A taxonomy of interdisciplinarity. The Oxford handbook of interdisciplinarity.

[CR77] Knaggård Å, Ness B, Harnesk D (2018). Finding an academic space: reflexivity among sustainability researchers. Ecol Soc.

[CR78] Lam JCK, Walker RM, Hills P (2014). Interdisciplinarity in sustainability studies: a review. Sustain Dev.

[CR79] Lapadat JC (2017). Ethics in autoethnography and collaborative autoethnography. Qual Inq.

[CR80] Levecque K, Anseel F, De Beuckelaer A (2017). Work organization and mental health problems in PhD students. Res Policy.

[CR81] Leverhulme Trust (2021) Leverhulme research centres. Available at: https://www.leverhulme.ac.uk/leverhulme-research-centres (accessed 12 Nov 2021)

[CR82] Lewis A (2021) Questioning the promise of interdisciplinarity: an ethnography of an interdisciplinary research project [PhD]. University of Nottingham

[CR84] Lindvig K (2018) The implied PhD student of interdisciplinary research projects within monodisciplinary structures. High Educ Res Dev 37(6):1171–1185

[CR85] Lindvig K, Hillersdal L (2019). Strategically unclear? Organising interdisciplinarity in an excellence programme of interdisciplinary research in denmark. Minerva.

[CR86] Louw J, Muller J (2014) A literature review on models of the PhD. Centre for Higher Education Transformation

[CR87] Lyall C (2019) Being an interdisciplinary academic: how institutions shape university careers. Palgrave Pivot

[CR88] Lynch S, Kuntz A (2019). ‘A critical autoethnography of a doctoral students’ research journey: learning to take risks in the academy’. Curric Stud Health Phys Educ.

[CR89] MacMynowski D (2007). Pausing at the brink of interdisciplinarity: power and knowledge at the meeting of social and biophysical science. Ecol Soc.

[CR90] Marelli S, Castelnuovo A, Somma A (2021). Impact of COVID-19 lockdown on sleep quality in university students and administration staff. J Neurol.

[CR91] McCann L, Granter E, Hyde P (2020). ‘Upon the gears and upon the wheels’: terror convergence and total administration in the neoliberal university. Manag Learn.

[CR92] Millar MM (2013). Interdisciplinary research and the early career: the effect of interdisciplinary dissertation research on career placement and publication productivity of doctoral graduates in the sciences. Res Policy.

[CR93] Mountford N, Coleman M, Kessie T (2020). Interdisciplinary doctoral research networks: enhancers and inhibitors of social capital development. Stud High Educ.

[CR94] Munro E, Heath S (2021) Falling short: response to UKRI’s phase 1 and phase 2 support for PhD researchers during the COVID-19 Pandemic. Pandemic PGRs

[CR95] Nurse P (2015). Ensuring a successful UK research endeavour: a review of the UK research councils.

[CR96] Oliver SK, Fergus CE, Skaff NK (2018). Strategies for effective collaborative manuscript development in interdisciplinary science teams. Ecosphere.

[CR97] O’Meara K, Culpepper D (2020). Fostering collisions in interdisciplinary graduate education. Stud Grad Postdr Educ.

[CR98] Phillips EM (2010). How to get a Phd: a handbook for students and their supervisors.

[CR99] Pischke EC, Knowlton JL, Phifer CC (2017). Barriers and solutions to conducting large international, interdisciplinary research projects. Environ Manag.

[CR100] Purvis B (2020) Operationalising urban sustainability: defining, measuring and modelling. [PhD]. University of Nottingham

[CR102] Quinn B (2020) Leading UK academics ask PhD funding body to rethink refusal of extra time. The Guardian, 17 Nov

[CR103] Rafols I, Leydesdorff L, O’Hare A (2012). How journal rankings can suppress interdisciplinary research: a comparison between Innovation Studies and Business & Management. Res Policy.

[CR104] Rings G (2021) The superdiverse precariat of British higher education? Limpiadores revisited. Curr Sociol 0011392120983345

[CR105] Robinson KF, Fuller AK, Stedman RC (2019). Integration of social and ecological sciences for natural resource decision making: challenges and opportunities. Environ Manag.

[CR106] Ross S, Savage L, Watson J (2020). University teachers and resistance in the neoliberal university. Labor Stud J.

[CR107] Russell Group (2016) Profile of the Russell group of universities. The Russell group of universities. Available at: https://russellgroup.ac.uk/media/5524/rg_text_june2017_updated.pdf (accessed 13 Oct 2022)

[CR108] Rylance R (2015). Grant giving: global funders to focus on interdisciplinarity. Nat News.

[CR109] Science Europe (2012). Science Europe position statement-horizon 2020: excellence counts.

[CR110] Science Europe (2017). Science Europe Position statement-on a new vision for more meaningful research impact assessment.

[CR111] Shen J (2020) Universities as financing vehicles of (sub)urbanisation: the development of university towns in Shanghai. Land Use Policy 112:104679

[CR112] Sousa SB (2011). The transformation of knowledge production and the academic community. Playing the game and (still) being an academic?. Educação, Sociedade e Culturas.

[CR113] Stanley P (2015). Writing the PhD journey(s): an autoethnography of zine-writing, angst, embodiment, and backpacker travels. J Contemp Ethnogr.

[CR114] Stichweh R (2003) Differentiation of scientific disciplines: causes and consequences. In: Encyclopedia of Life Support Systems (EOLSS). UNESCO, Paris

[CR116] Tripathi A, Polus R, Zhang Y, Nautiyal R, Shaheer I (2022). ‘Remember that time?’: Introducing retrospective collaborative autoethnography. Tour Recreat Res.

[CR117] Tucker BP, Parker LD, Merchant KA (2016). With a little help from our friends: an empirical investigation of co-authoring in accounting research. Br Account Rev.

[CR118] Trussell DE, Paterson S, Hebblethwaite S (2017). Negotiating the complexities and risks of interdisciplinary qualitative research. Int J Qualit Methods.

[CR119] UKRI (2021) Impact toolkit for economic and social sciences. UKRI

[CR120] University and College Union (2012) Choice cuts: how choice has declined in higher education. University and College Union

[CR121] Urban ECA Collective, Ahmed N, Baker AG, Bhattacharya A, Cawood S, Cabrera Pacheco AJ, Daniel MM, Grandi M, Grimaldo-Rodríguez CO, Guma PK, Habermehl V, Higgins K, Lata LN, Liu M, Luederitz C, Macktoom S, Macrorie R, Melgaço L, Morales I, Westman L (2022) Redefining the role of urban studies Early Career Academics in the post-COVID-19 university. City 26(4):562–586. 10.1080/13604813.2022.2091826

[CR122] Vernon J (2018). The making of the neoliberal university in Britain. Crit Hist Stud.

[CR123] Villeneuve D, Durán-Rodas D, Ferri A (2020). What is interdisciplinarity in practice? Critical reflections on doing mobility research in an intended interdisciplinary doctoral research group. Sustainability.

[CR124] Viseu A (2015). Caring for nanotechnology? Being an integrated social scientist. Soc Stud Sci.

[CR125] Wagner CS, Roessner JD, Bobb K (2011). Approaches to understanding and measuring interdisciplinary scientific research (IDR): a review of the literature. J Informetr.

[CR126] Wall S (2008). Easier said than done: writing an autoethnography. Int J Qualit Methods.

[CR127] Wang C, Zhao H (2020) The Impact of COVID-19 on anxiety in Chinese university students. Front Psychol 11:1168 10.3389/fpsyg.2020.01168 PMC7259378 32574244

[CR128] Weingart P (2010). A short history of knowledge formations. The Oxford handbook of interdisciplinarity.

[CR129] Wilson A, Reay D, Morrin K (2021). ‘The still-moving position’ of the ‘working-class’ feminist academic: dealing with disloyalty, dislocation and discomfort. Discourse.

[CR130] Wray S, Kinman G (2021). Supporting staff wellbeing in higher education.

